# Mathematically modeling worried-well behavior during infectious disease outbreaks

**DOI:** 10.1371/journal.pone.0319550

**Published:** 2025-09-29

**Authors:** Bismark Singh, Dmitry Gromov

**Affiliations:** 1 School of Mathematical Sciences, University of Southampton, Southampton, United Kingdom; 2 Department of Mathematics, University of Latvia, Rīga, Latvia; Caleb University, NIGERIA

## Abstract

Although curtailing pathogen spread is critical for mitigating the impact of novel infectious disease outbreaks, addressing the psychological and social responses of populations is also important. This is because uninfected individuals who display an excessive concern of the disease can significantly strain healthcare systems. In existing research, the transmission dynamics of such “worried-well” behavior is largely unexplored. We present a mathematical modeling framework to study such spread alongside the pathogen’s transmission. Our approach extends traditional compartmental models to specifically include the psychological transmission of worry, while acknowledging two extremes of this behavioral response: overly cautious and defiantly protesting. We provide guidance for policymakers, towards healthcare resource allocation and disease outbreak management, by deriving insights into the differential impacts of both these behaviors. Our findings suggest that different strategies are required to manage worried-well surges, depending on the dominant behavioral regime.

## 1 Introduction

Novel infectious disease outbreaks, caused by previously unidentified strains, are characterized by uncertainty. This uncertainty manifests itself both in the spread of the inducing pathogen, and in the evolution of public perception plus its corresponding behavioral responses [1]. Although there is extensive epidemiological research on modeling the spread of infectious disease—such as, determining the trajectories of symptomatic and asymptomatic infections [2]—the parallel spread of concern and its associated behavioral response is lesser studied. An adequate understanding of such behavioral dynamics is critical for effective outbreak management for two key reasons.

First, public perception of the pandemic directly influences compliance towards or away from disease control measures. The resulting behavioral changes—even in uninfected individuals—can significantly affect the trajectory of the pathogen’s spread, see, e.g., [3,4]. An example of such a behavioral change is social distancing. A significant amount of evidence from the COVID-19 pandemic showed that following social distancing mandates reduced the pathogen’s spread [5]. However, adherence to social distancing measures varied widely as determined by an individual’s risk perception of the outbreak [6]. Some individuals responded with elevated levels of anxiety by avoiding social contact excessively [7], while others rejected public health guidelines by protesting against unrestrained governmental interventions [8]. These responses necessitate distinct mitigation strategies, presenting separate challenges for policymakers.

Second, reliable asymptomatic testing is often unavailable at the outbreak’s onset [9]. Due to the absence of clear diagnostic tools, healthcare professionals may struggle to distinguish genuinely infected individuals from those exhibiting worried-well behavior [10,11]. The term *worried-well* refers to individuals who exhibit significant anxiety about a disease outbreak—despite being clinically uninfected—and alter their behavior. This diagnostic ambiguity risks an inefficient allocation of scarce therapeutic resources which may lead to shortages for those genuinely in need. This risk is particularly severe if effective vaccines or countermeasures are not yet widely available. Thus, accurate estimates of worried-well individuals are essential for determining overall demand of resources, as well as fair and effective allocation policies.

The concept of worried-well has roots within the medical and psychiatric literature. Originally, it referred to patients who presented significant health concerns despite the absence of clear physical symptoms. For example, the term *syphilophobia* was coined to describe a fear of syphilis infection without any clinical evidence of the disease [12]. Similar anxiety-driven health concerns emerged during the AIDS epidemic of the late 1980s as uninfected individuals sought frequent medical reassurance [13]. However, the formal recognition of the worried-well as a distinct patient category emerged in the 1970s when health maintenance organizations (HMOs) began classifying incoming patients into three groups: well, asymptomatically sick, and truly sick [14]. This early classification recognized that portions of the healthcare-seeking population might be motivated by psychological distress rather than pathogen-induced illness.

More recent studies refined this classification identifying three primary subgroups within the worried-well population: (a) individuals experiencing disease symptoms due to anxiety, but without pathogen exposure, (b) individuals working in high-risk activities seeking reassurance of their concerns, but without any symptoms of the pathogen, and (c) those with anxiety following a traumatic event [15,16]. At an individual level, such concerns often arise from anxiety, fear, or distress. At a social level, they can spread rapidly through “mass psychogenic illnesses” triggered by factors such as unusual smells [17,18], vaccine campaigns [19], or catastrophic events [20].

Understanding these psychological dynamics is critical for designing effective public health responses, especially in the early stages of an outbreak. Traditional epidemiological models focus primarily on pathogen transmission; however, a parallel framework is needed to capture the spread of fear and anxiety within the uninfected population. Our work stems from this motivation and seeks to both quantify worried-well behavior and develop practical guidance for managing it. With this background, the primary aim of our work is to provide quantitative guidance for healthcare policymakers on how to manage surges in worried-well populations during the early days of a pathogen-induced infectious disease outbreak. The following are the two key contributions of this article.

(a) We present a mathematical framework to estimate worried-well populations in a pathogen-induced disease outbreak. Our model captures a spectrum of behavioral responses, ranging from overly cautious self-isolation to active protest against public health mandates.
(b) We provide empirical guidance for policymakers on therapeutic resource allocation during the critical early stages of an outbreak, when information on both pathogen spread and public concern is limited. Specifically, we analyze two contrasting policy approaches: conserving scarce medical resources for later phases versus distributing them immediately to alleviate public pressure.

The rest of this article is organized as follows. In Sect 2, we describe the compartmental model and its parameters. Sect 3 presents our main results, and their implications for public health policy. Sect 4 concludes with a summary and limitations of our work.

## 2 Materials and methods

We consider a pathogen-induced infectious disease outbreak, where both worried-well and pathogen-infected populations evolve over time. The spread of worried-well behavior is analogous to infectious disease transmission, and both processes propagate independently through contacts with correspondingly infected individuals. However, unlike a pathogen that transmits through physical proximity or direct contact, worry can also spread through less tangible ways such as public health messaging [21], governmental campaigns [22], propaganda [23], and online social networks [24]. In this sense, we are motivated by the basic two-disease model of Blyuss and Kyrychko [25]. Table 1 summarizes the notation used in this work.

**Table 1:** State variables and parameters for model Eq (1).

Parameter	Description	Units
Compartments		
*S*	fraction of individuals susceptible to both pathogens and worry	—
*I*_*P*_	fraction of individuals infected by a pathogen who may or may not be worried (i.e., genuinely sick)	—
*I*_*W*_	fraction of individuals worried but not infected by a pathogen (i.e., worried-well)	—
*R*_*P*_	fraction of individuals recovered from the pathogen infection	—
Parameters		
*α*	behavioral modifier for the worried-well, $\alpha \in {\mathbb{R}}^{+}$	
$\beta_{P}$	transmission coefficient of pathogen	[day^−1^]
$\beta_{W}$	transmission coefficient of worried-well	[day^−1^]
$\beta_{WP}$	transmission coefficient of worry from contact with a sick individual	[day^−1^]
$\gamma_{P}$	transition rate of recovery from pathogen	[day^−1^]
$\gamma_{W}$	transition rate of recovery from worry	[day^−1^]
$\delta_{P}$	transition rate at which individuals recovered from pathogen become susceptible again	[day^−1^]

We divide the host population, *N*, into three primary compartments: susceptible (*S*), infected (*I*), and recovered (*R*). Given the short time scales typically involved in early outbreak dynamics, we assume negligible mortality and ignore demographic changes; thus, the total population is constant. We consider the following compartments of the total population:

- Susceptible (*S*): containing individuals who have not yet been infected by the pathogen and are also not currently worried.
- Pathogen-Infected (*I*_*P*_): Individuals who are genuinely sick due to an infection by the pathogen, irrespective of their psychological state. These individuals can recover through medical intervention (e.g., antivirals or antibiotics), social distancing, or rest.
- Worried-Well (*I*_*W*_): Individuals who are uninfected by the pathogen but alter their behavior due to a perceived risk of infection.
- Recovered (*R*): Individuals who have recovered from the pathogen, gaining temporary immunity to both the pathogen and worry.

We assume recovery from the pathogen confers temporary immunity to both the pathogen and worry, placing individuals in the *R*_*P*_ compartment. In contrast, recovery from worry alone does not provide immunity; thus, individuals may re-enter the worried-well compartment upon subsequent exposure. This assumption is motivated by previous studies on recurrence of anxiety disorders [29].

**Fig 1:**
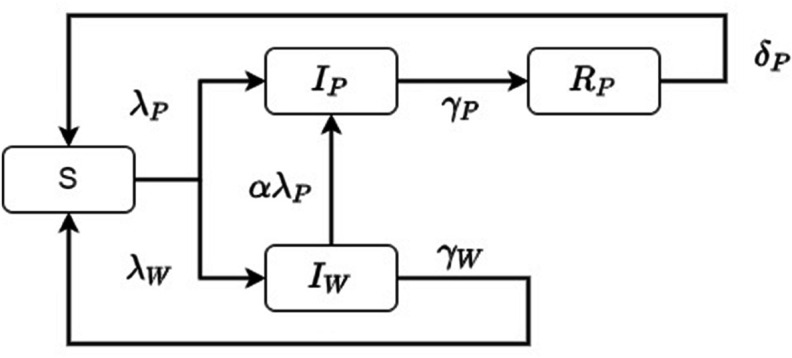
Flow diagram of the two-process compartmental model (1). The arrows indicate epidemiological or psychological transitions between the different compartments, while the Greek letters provide the corresponding rates.

The spread of pathogen is governed by contacts with individuals in the *I*_*P*_ compartment alone. However, worried-well behavior spreads via contacts of two types: those with individuals in the compartment *I*_*P*_, and those with individuals in the compartment *I*_*W*_. Thus, as shown in Fig 1, individuals enter the *I*_*P*_ compartment from both the *S* and *I*_*W*_ compartments, while they enter the *I*_*W*_ compartment from *S* alone. We denote the transmission coefficients for these three types of contacts by $\beta_{P}$, $\beta_{WP}$, and $\beta_{W}$, respectively. We then have the two forces of infection (see, e.g., [26]) defined as follows: $\lambda_{P}=\beta_{P}I_{P}$ and $\lambda_{W}=\beta_{WP}I_{P}$  +  $\beta_{W}I_{W}$. Analogously, we let $\gamma_{P}$ and $\gamma_{W}$ denote the transition rates corresponding to the removal of the pathogen and worry, respectively. These transition rates equal the inverse average duration of the respective conditions, while $\delta_{P}$ is the inverse average duration of the immunity to the pathogen.

As we mentioned in Sect 1, worried-well behavior results in an altered lifestyle for these individuals. However, these concerns are genuinely distinguished from those of the pathogen-infected population, who often reduce contacts as a protective measure, altering their lifestyle in response to physical illness [27,28]. This distinction informs our choice to introduce a separate behavioral factor, α>0, for the worried-well:

(a) *cautious* individuals who excessively decrease their contacts have α<1, and
(b) *protesting* individuals who excessively increase their contacts (for instance, by attending mass events and meetings) have α>1.

The default state where individuals are simply worried but do not alter their behavior is modeled via α=1. This behavioral factor changes the force of infection, $\lambda_{P}$, from the worried-well to the pathogen-infected compartments; thus, it influences the overall dynamics of the outbreak.

Since the total population is constant, we formulate the model in terms of the fractions of the total population. This implies that all system states belong to the interval [0,1] and the sum of the variables equals 1, i.e., $S+I_{P}+I_{W}+R_{P}=1$. With this background, we have the following epidemiological compartmental model:

$$\frac{\text{d}S}{\text{d}t}=-(\beta_{P}+\beta_{WP})SI_{P}-\beta_{W}SI_{W}+\delta_{P}R_{P}+\gamma_{W}I_{W},$$ (1a)

$$\frac{\text{d}I_{P}}{\text{d}t}=\beta_{P}SI_{P}+\alpha \beta_{P}I_{P}I_{W}-\gamma_{P}I_{P},$$ (1b)

$$\frac{\text{d}I_{W}}{\text{d}t}=-\alpha \beta_{P}I_{P}I_{W}+\beta_{W}SI_{W}+\beta_{WP}SI_{P}-\gamma_{W}I_{W},$$ (1c)

$$\frac{\text{d}R_{P}}{\text{d}t}=\gamma_{P}I_{P}-\delta_{P}R_{P}.$$ (1d)

Eqs (1a)–(1d) describe the dynamics of the four compartments presented in Fig 1. Then, the basic reproduction number for model (1) computed using the next-generation matrix method (see, e.g., [30]) equals the maximum of the two respective reproduction numbers (see, e.g., [25]):

$$R_{0}=\max\left\{\frac{\beta_{P}}{\gamma_{P}},\frac{\beta_{W}}{\gamma_{W}}\right\}.$$ (2)

If *R*_0_>1, disease, worry, or both become endemic in the population. Although the reproduction number is important in determining the expected number of infections at the steady-state, our work is focused on the early-stage transient dynamics of the disease.

Interestingly, the reproduction number is independent of $\beta_{WP}$. Intuitively, this is so because individuals infected by the pathogen are already effective vectors for worry. To illustrate this formally, consider the disease-free equilibrium $\left[S^{*},\;I_{P}^{*},\;I_{W}^{*},\;R_{P}^{*}\right]$ with the corresponding disease free steady state [1,0,0,0]. Then, as described in [25], linearizing the system of equations in model (1) for the three independent variables $\left[I_{p},\;I_{W},\;R_{P}\right]$ near the disease free steady state provides the following matrix (note that *S* is obtained from $S+I_{P}+I_{W}+R_{P}=1$).


$$[\begin{array}{ccc} \beta_{P}-\gamma_{P} & 0 & 0\\ \beta_{WP} & \beta_{W}-\gamma_{W} & 0\\ \gamma_{P} & 0 & -\delta_{P} \end{array}].$$

All eigenvalues of this matrix must be negative to ensure stability of the disease-free equilibrium state. The three eigenvalues are $\beta_{P}-\gamma_{P},\beta_{W}-\gamma_{W}$ and-$\delta_{P}$. The third eigenvalue is trivially negative, while the requirement of negativity of the first two provides the condition *R*_0_<1 for the basic reproduction number given by Eq (2).

The parameters we use in our numerical experiments are informed by early estimates from the COVID-19 pandemic. We use $\gamma_{P}=\gamma_{W}=\frac{1}{14}$ day^−1^ following [31] where the estimated mean time from the onset of symptoms to two negative RT-PCR tests is taken as two weeks. We assume worry to persist equally long, on average, as the pathogen. We consider the basic number of reproductions of the pathogen-induced disease, $\frac{\beta_{P}}{\gamma_{P}}$, as 10.3 after [32]; then, $\beta_{P}=0.74$ day^−1^. We consider a conservative estimate of the average duration of pathogen immunity as 8 months following [33]; thus, $\delta_{P}=\frac{1}{8\times 30}$ day^−1^. We are unaware of any studies providing estimates of the transmission rate or the basic reproduction number for worry. We consider $\beta_{W}=\beta_{WP}=0.7$ day^−1^; i.e., the reproduction number of the worry-induced disease is 9.8, which is slightly less than the reproduction number of the pathogen-induced disease. The default initialization of our simulations described in Sect 3 assumes 1% of the total population is genuinely infected and another 1% is worried but uninfected.

## 3 Results and analysis

In the absence of asymptomatic testing, the quantity *I*_*P*_  +  *I*_*W*_ is the best proxy for the total burden on the healthcare system. However, this does not directly equate to the number of individuals seeking therapeutic resources, as some worried-well individuals (*I*_*W*_) may intentionally avoid healthcare settings (we explain this below). Individuals without symptoms were explicitly labeled as a non-priority testing group as of March 22, 2020, by the US Centers for Disease Control and Prevention (CDC) [34]. Thus, during the early stages of a novel disease outbreak, the consumption of scarce resources by the worried-well can deprive genuinely infected individuals (*I*_*P*_). The ratio $\frac{I_{W}}{I_{P}}$ serves as one indicator of the public pressure that the worried-well population imposes on healthcare policymakers.

When this ratio exceeds one and asymptomatic testing is unavailable, resource allocation policies should seek to reduce the disproportionately large worried-well population. Then, an advisable policy is to conserve resources anticipating increased demand from genuinely infected individuals at a later stage of the outbreak. Conversely, when the ratio is less than one, the primary focus should be on reducing pathogen spread. Then, a more immediate allocation of available resources, despite scarcity, is advisable, as the worried-well can be effectively treated as susceptible individuals. For further discussions on immediate and sequential release policies, see [35].

We begin by analyzing the default behavioral case of α=1 whose evolution is shown in Fig 3 (see, also Fig 5). The outbreak begins with worry surpassing infection: during the first few days, we have $I_{W}>I_{P}$. This mirrors early-February 2020 when many countries recorded almost no infected COVID-19 cases; however, they reported large amounts of worried-well cohorts [1]. In approximately two weeks, *I*_*P*_ peaks and exceeds *I*_*W*_. Following this, worry starts declining due to the onset of pandemic fatigue [36]; we model this via the recovery rate, $\gamma_{W}$. True infections continue to rise because both susceptible and worried-well individuals acquire the disease. This divergence showcases the distinct dynamics governing psychological versus biological contagion.

**Fig 2:**
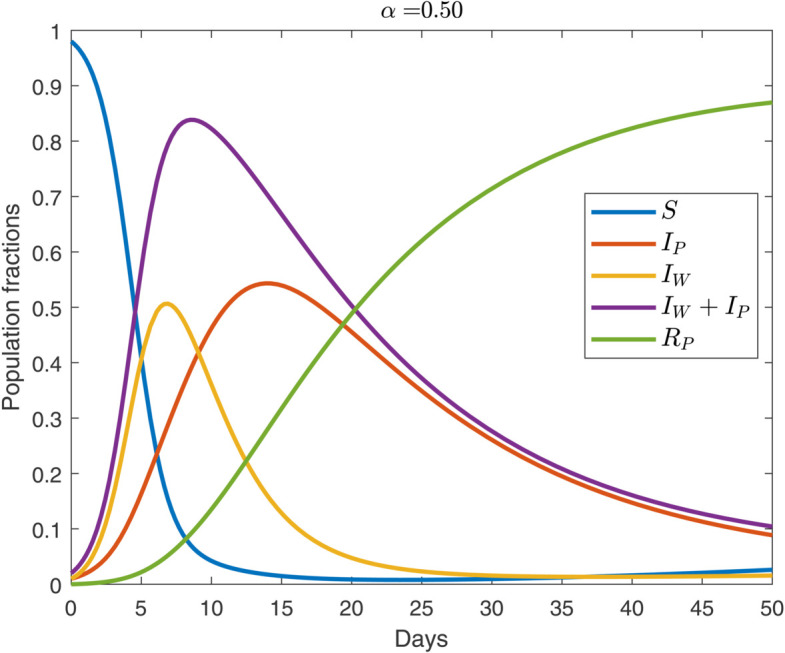
Temporal propagation of pathogen-induced disease and worried-well behavior over 50 weeks for the cautious regime (α<1). For the parameter values, see Sect 2. The initial condition is $I_{P}=I_{W}=0.01$.

**Fig 3:**
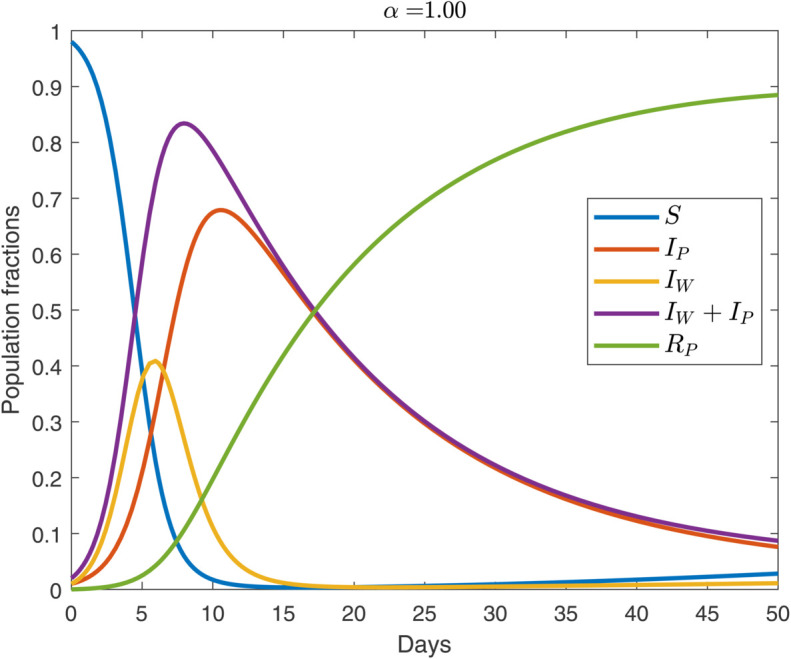
Temporal propagation of pathogen-induced disease and worried-well behavior over 50 weeks for the default regime (α=1). For the parameter values, see Sect 2. The initial condition is $I_{P}=I_{W}=0.01$.

**Fig 4:**
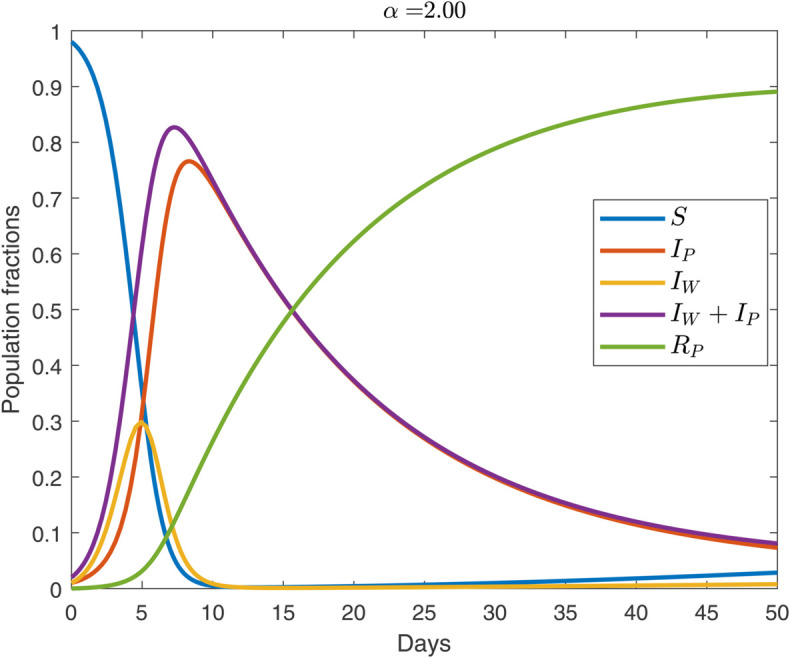
Temporal propagation of pathogen-induced disease and worried-well behavior over 50 weeks for the protesting regime (α>1). For the parameter values, see Sect 2. The initial condition is $I_{P}=I_{W}=0.01$.

**Fig 5:**
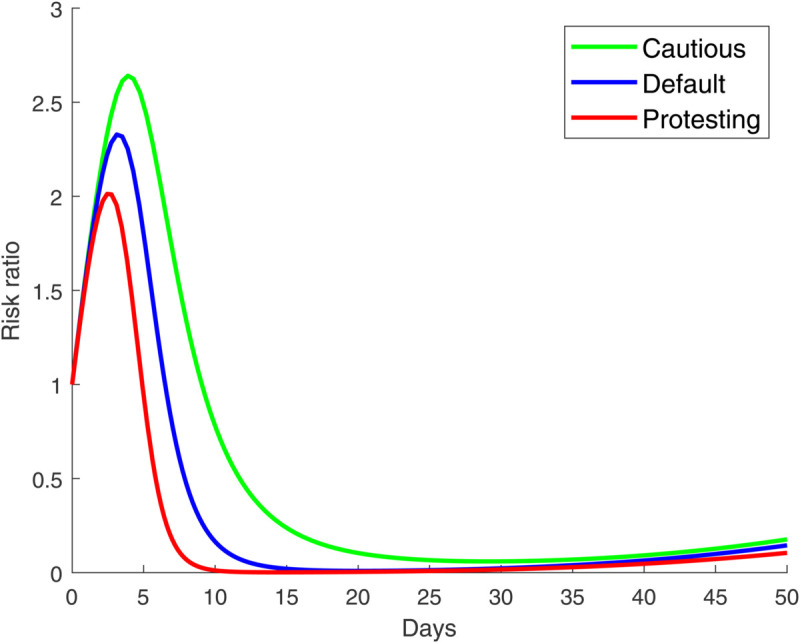
Ratio of worried-well to genuinely infected individuals ($\frac{I_{W}}{I_{P}})$ for the cautious (α<1), default (α=1), and protesting (α>1) regimes. Values above 1 indicate that perceived demand (driven by worry) exceeds the actual infectious demand.

Controlling the two components of the disease outbreak requires two separate strategies. Standard and well-studied pathogen control strategies—mask mandates, social-distancing, vaccination, etc.—target transmission among the genuinely infected populations. In contrast, limiting the *inflow* of worried-well individuals into the infected pool requires a reduction in their contact rates alone. We capture these behavioral shifts via the parameter *α*: cautious responses that dampen contacts (α<1) and protesting responses (α>1) that amplify contacts. Next, we analyze both of these regimes.

Effective public health messaging can shift behavior towards the cautious regime (i.e., α<1) by increasing compliance in the uninfected population [3]. Past work shows that individuals who rely on reputable news sites maintain stricter social distancing than those who receive information from social-media feeds [3,39]. Fig 2 (see, also Fig 5) illustrates this scenario: the peak of genuinely infected individuals (*I*_*P*_) is lower than the default case (see, Fig 3), yet the overall demand for resources ($I_{P}+I_{W}$) is nearly the same. This is because although cautious behavior suppresses the spread of the pathogen, it simultaneously increases the pool of the worried-well population; i.e., the ratio $\frac{I_{W}}{I_{P}}$ is driven upward. This pattern can lead to dramatic headlines and media coverage even when the actual number of infections is declining. For example, New York City faced surges of consultations from the worried-well populations patients during the 2009 H1N1 pandemic [37]. Political dynamics can amplify this pressure; e.g., during the Ebola outbreak, Republicans opposed the US federal government’s response, whereas during the Zika outbreak, the Democrats blocked the Republican response [38].

Public pressure on healthcare policymakers within the cautious regime may be defused following the availability of diagnostic asymptomatic testing. Then, clinicians can distinguish truly infected patients (*I*_*P*_) from worried– well individuals (*I*_*W*_), revealing that actual risk (*I*_*P*_) is lower than the headline figure (*I*_*P*_  +  *I*_*W*_). At this stage, public-facing measures that calm anxiety—such as, reassuring social-media messaging [40] or community rituals that restore a sense of control [41]—are advisable as they help shift *I*_*W*_ back into the susceptible pool. Ultimately, this eases pressure on hospitals without loosening core infection-control rules. Importantly, such reassurance strategies complement—rather than replace—social distancing, and other pathogen-focused interventions because the remaining susceptible population is still at risk.

Now, consider the protesting regime (α>1) where worry amplifies pathogen transmission by driving individuals toward contact-rich settings. Such behavior manifests itself either through mass demonstrations against public-health mandates [8] or frequent reassurance-seeking hospital visits [16]. Despite the very different motives (defiant skepticism versus anxious reassurance), both behaviors increase effective contact rates, pushing worried-well individuals (*I*_*W*_) rapidly into the infected pool. The result is that this regime produces the largest peak of genuinely infected individuals (*I*_*P*_) among all three behavioral settings, see, Fig 4 (see, also Fig 5). We note that the early phase of the outbreak is still dominated by worry; yet, infections quickly exceed worried-well counts due to the rapid increase in contact rates. This dynamic can overwhelm healthcare systems, especially if paired with public mistrust in government interventions.

In the protesting regime, the peak demand for resources (at most *I*_*P*_  +  *I*_*W*_) arrives sooner than in either the cautious or default settings, presenting a dual challenge for public health officials. First, the rapid onset leaves less time for system surge preparation. Second, the perceived risk is close to the actual clinical burden, since the observed demand is almost entirely from the genuinely infected individuals. This is true even under optimistic assumptions of early asymptomatic testing—which is unlikely to be widely available given the novelty of the pathogen. To manage this combined threat, policymakers must simultaneously suppress pathogen transmission and address the psychological drivers of worry. One approach is to enforce strict early lockdowns that limit contact across the entire population, thus dampening both pathogen and worry spread simultaneously [42]. However, such authoritarian measures can provoke backlash, including larger protests once restrictions are lifted [8], as well as heightened public anxiety and depression [43]. An alternative is to release scarce therapeutics and diagnostics early, without holding them back for future waves [35]. In this high-contact regime, the risk of wastage is low because few uninfected individuals remain to consume those resources; further, early access may also help reassure anxious patients [44]. However, this strategy carries its own uncertainty, as it remains unclear whether worried-well individuals who distrust government policy would view such an approach as genuine support or as a tactic to suppress dissent.

The analysis so far assumes that the initial sizes of the worried-well and genuinely infected populations are identical (both comprising 1% of the total population). To test the sensitivity of our results to this choice, we conduct additional simulations with asymmetric initial conditions (see Appendix). We find that the qualitative dynamics remain largely unaffected, although the severity of the outbreak (i.e., the number of worried-well or pathogen-infected populations) varies with the initial population fractions. For example, even when the initial worried-well population is ten times smaller than the genuinely infected population, the general progression of the two groups remains similar (see, S1 Fig in the Appendix), and the peak value of the ratio $\frac{I_{W}}{I_{P}}$ still exceeds two (see, S2 Fig in the Appendix). In contrast, this peak value surpasses 2.5 when the initial proportions are balanced, reflecting a more sustained dominance of worry early in the outbreak. The reverse setting—where the initial worried-well population is ten times larger than the genuinely infected population—produces a greater imbalance, although the overall trend still remains the same. Here, the peak value of $\frac{I_{W}}{I_{P}}$ is nearly 10 (see, S2 Fig), underscoring the self-reinforcing nature of worry in the absence of early containment. This result is consistent with the high basic reproduction numbers (*R*_0_) used in our simulations, due to which both worry and infection spread rapidly at the start, and largely dominate the effects of the initial conditions.

## 4 Conclusions

Public concern can surge long before a pathogen achieves widespread transmission, thereby rapidly straining healthcare capacity and emergency services with reassurance-seeking visits. Empirical reports suggest that worried-well consultations may exceed true infections by up to twenty-fold, making them one of the most demanding challenges of outbreak management [45]. For example, in February 2020, Canada recorded only four confirmed COVID-19 cases, yet 2.6 million people reported being “very concerned” [1]. After the 1995 Tokyo sarin attacks, nearly three-quarters of incoming healthcare visits required no medical treatment [20]. Although such worry is rarely malicious, it can paradoxically increase transmission via nosocomial exposure risk, as our model shows.

This challenge is most acute during the early, information-scarce phase of a novel epidemic, when *perceived* risk diverges sharply from *actual* risk. Since perceived risk of the outbreak directly influences preventative behavior [46], an estimation of public anxiety is essential. In the absence of such data, risk-seeking decision-makers may underestimate the worried-well and allow facilities to be overrun, whereas risk-averse leaders may overuse scarce assets, incurring unnecessary economic costs. Historical responses illustrate this spectrum: President Franklin Roosevelt dramatically expanded US polio funding, and President Xi Jinping imposed stringent COVID-19 lockdowns, both of which are high-risk and resource-intensive strategies. In contrast, Chancellor Angela Merkel maintained a restrained stance during the Ebola threat, and Prime Minister Manmohan Singh took limited action against the 2009 H1N1 outbreak, exemplifying risk-averse approaches. Since political leaders often act on perceived rather than actual risk, any exaggerated public perceptions can directly influence these decisions.

The World Health Organization’s Director-General warned on 15 February 2020 that “We’re not just fighting an epidemic; we’re fighting an infodemic. Fake news spreads faster and more easily than this virus, and is just as dangerous.” [47]. Our analysis substantiates this concern: even a small trace of anxiety can trigger a self-reinforcing surge of worried-well individuals whose numbers, and corresponding demand for reassurance, may outweigh those who are truly infected. Without timely estimates and data on this behavioral spread, authorities risk misallocating scarce clinical resources and, thus, amplifying public fear. Our results suggest that until reliable diagnostics and vaccines are not available, a conservative policy of maintaining a safeguarded cache of therapeutics offers the best hedge against both epidemiological and psychological uncertainty. Following the widespread availability of such medical countermeasures, the genuinely infected population is expected to exceed the worried-wells.

Although our model captures the details of the early-stage progression of the worried-well behavior and its interaction with the pathogen spread, there are several simplifying assumptions which may be refined in future work. For example, we assume identical recovery rates for worry and infection ($\gamma_{W}=\gamma_{P}$); this reflects the practical challenge of lacking reliable psychological data from previous disease outbreaks. This assumption is reasonable as a first approximation, since both physical recovery from mild viral infections and psychological recovery from acute anxiety often occur on comparable timescales (e.g., 1–2 weeks) [31]. However, the resurgence or persistence of worry differs based on both an individual and social context, as well as media influence, suggesting future models should consider distinct recovery rates.

Additionally, our current framework does not explicitly account for feedback loops where sustained worry influences both public behavior and policy responses, potentially creating self-reinforcing cycles of fear and mistrust. Capturing this nuance requires more sophisticated mathematical models that integrate social-media dynamics, risk perception thresholds, and long-term psychological effects. Finally, our model also assumes worry spreads similar to a pathogen; however, the psychological contagion of fear may follow different social dynamics, potentially requiring a fundamentally different mathematical modeling approach.

To conclude, surges of worry are not only a clinical challenge but also one of communication. Dampening such surges requires a tiered strategy: clear, evidence-based messaging for all groups and tailored interventions for those at heightened psychological risk (including both ends of the spectrum of worried-well). Ultimately, this requires building public trust in institutions, which is the hallmark of any successful health-communication campaign [48].

## Supporting information

S1 Appendix Sensitivity to asymmetric initial conditions. (PDF)
