# Supplementary material for: Mathematically modeling worried-well behavior during infectious disease outbreaks
Source: PLoS One. 2025 Sep 29;20(9):e0319550. doi: 10.1371/journal.pone.0319550 (PMC12478904; doi:10.1371/journal.pone.0319550)
Supplement: S1 Appendix — (PDF) [file pone.0319550.s001.pdf]

Supplementary Information for: Mathematically modeling worried-well behavior during infectious disease outbreak

Bismark Singh[a,*], Dmitry Gromov[b]

[a]*School of Mathematical Sciences, University of Southampton, Southampton, UK*
[b]*Department of Mathematics, University of Latvia, Rīga, Latvia*

Our results in the main text consider an equal fraction of the worried-well and the genuinely infected population (both 1% of the total population). To assess the robustness of our findings, we conduct additional simulations with varying initial population fractions. Specifically, we consider two cases, distinguished by extremes of the initial cases, where the initially worried population is ten times less, or more, than the initially infected population. In this appendix, we provide additional plots complementing those in the main text: Fig. S1 is analogous to Fig. 2 of the main text, while Fig. S2 is analogous to Fig. 3 of the main text.

*Corresponding author
Email address: b.singh@southampton.ac.uk (Bismark Singh)

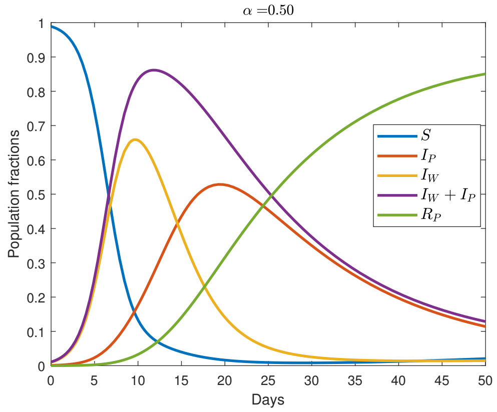

(a) Cautious behavior

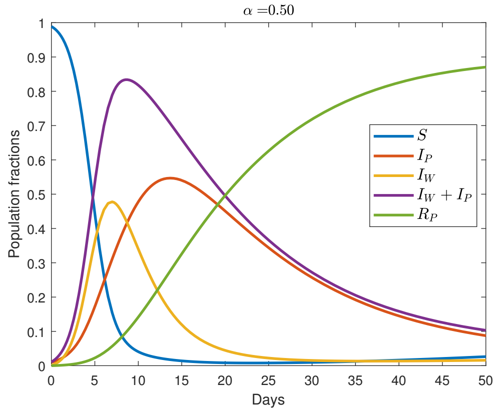

(b) Cautious behavior

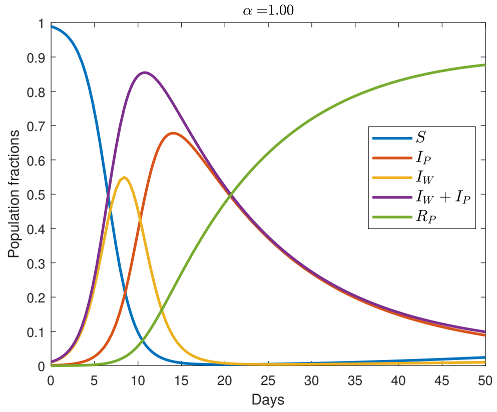

(c) Default behavior

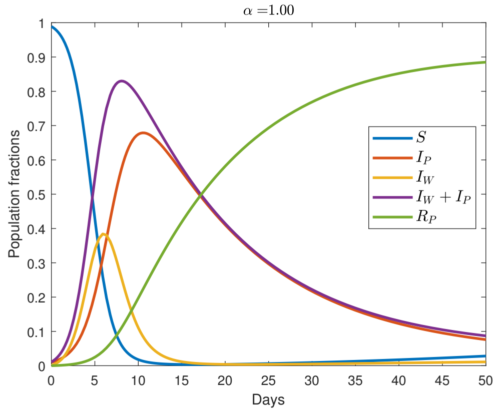

(d) Default behavior

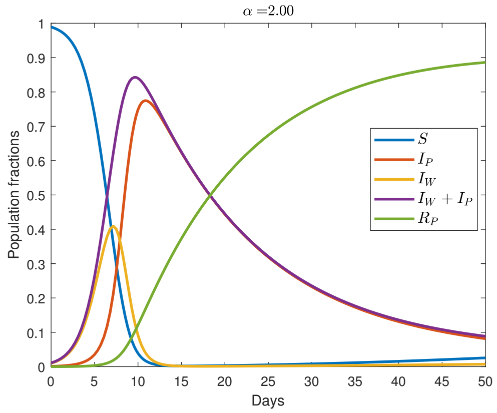

(e) Protesting behavior

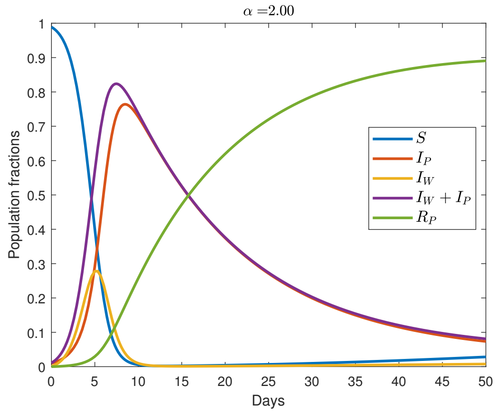

(f) Protesting behavior

Figure S1: Sensitivity to asymmetric initial conditions: analogous graphs to Fig. 2 of the main text. In the left panel, the initial condition is $I_P = 0.001, I_W = 0.01$ (worried-well predominates); while, in the right panel, the initial condition is $I_P = 0.01, I_W = 0.001$ (pathogen predominates).

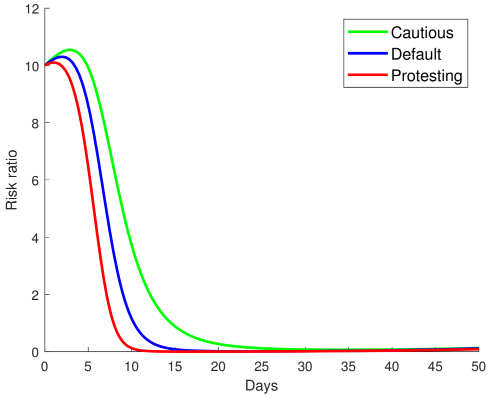

(a)

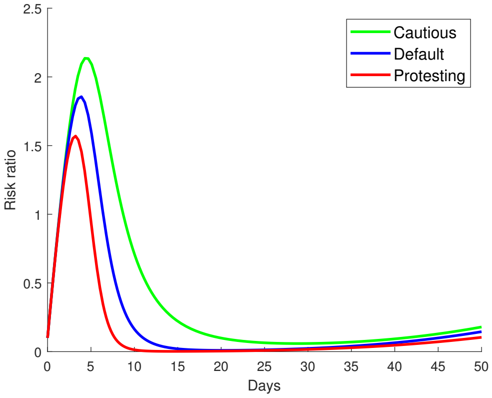

(b)

Figure S2: Sensitivity to asymmetric initial conditions: analogous graphs to Fig. 3 of the main text. In the left panel, the initial condition is $I_P = 0.001, I_W = 0.01$ (worried-well predominates); while, in the right panel, the initial condition is $I_P = 0.01, I_W = 0.001$ (pathogen predominates).
